# Supplementary material for: Melamine Foam-Derived Carbon Scaffold for Dendrite-Free and Stable Zinc Metal Anode
Source: Molecules. 2023 Feb 11;28(4):1742. doi: 10.3390/molecules28041742 (PMC9964734; doi:10.3390/molecules28041742)
Supplement: Supplementary file 1 [file molecules-28-01742-s001.zip › molecules-2101150-supplementary.pdf]

molecules

Supporting Information

Melamine Foam-Derived Carbon Scaffold for Dendrite-Free and Stable Zinc Metal Anode

Yong Liu [1,2,*,†], Feng Tao [1,†], Yibo Xing [1], Yifei Pei [1] and Fengzhang Ren [1,3,*]

[1] School of Materials Science and Engineering, Provincial and Ministerial Co-Construction of Collaborative Innovation Center for Non-Ferrous Metal New Materials and Advanced Processing Technology, Henan University of Science and Technology, Luoyang 471023, China
[2] Science & Technology Innovation Center for Advanced Materials of Intelligent Equipment, Longmen Laboratory, Luoyang 471023, China
[3] Henan Key Laboratory of Non-Ferrous Materials Science & Processing Technology, School of Materials Science and Engineering, Henan University of Science and Technology, Luoyang 471023, China
* Correspondence: liuyong209@haust.edu.cn (Y.L.); renfz@haust.edu.cn (F.R.)
† These authors contributed equally to this work.

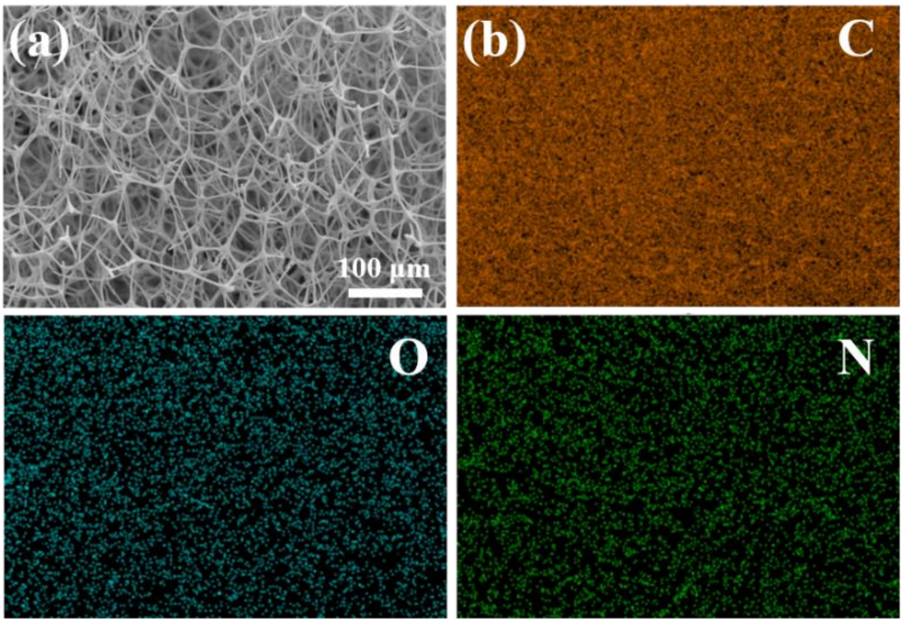

Figure S1. (**a**) SEM and (**b**) corresponding mapping images of CF.

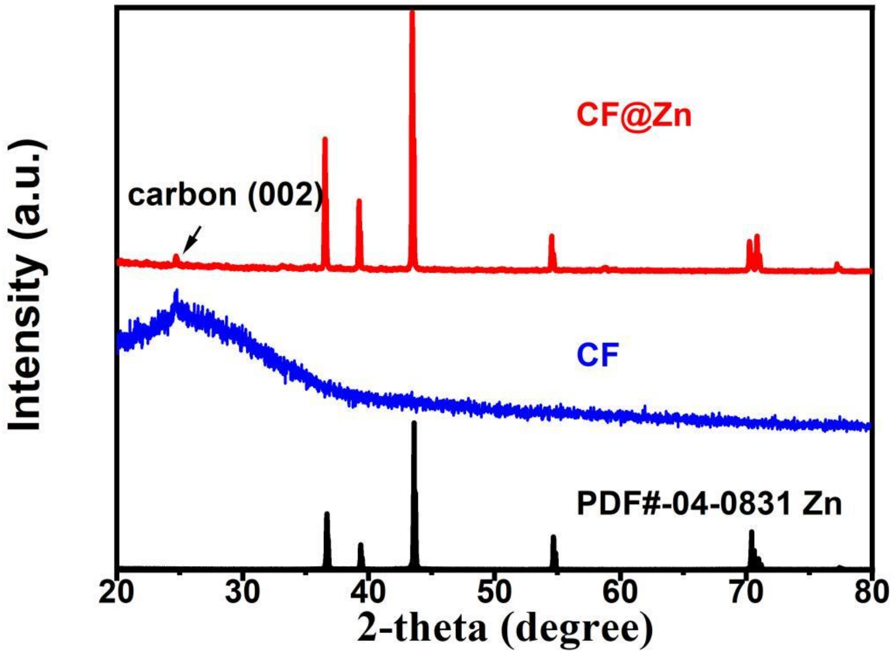

Figure S2. XRD patterns of pure CF and CF@Zn samples.

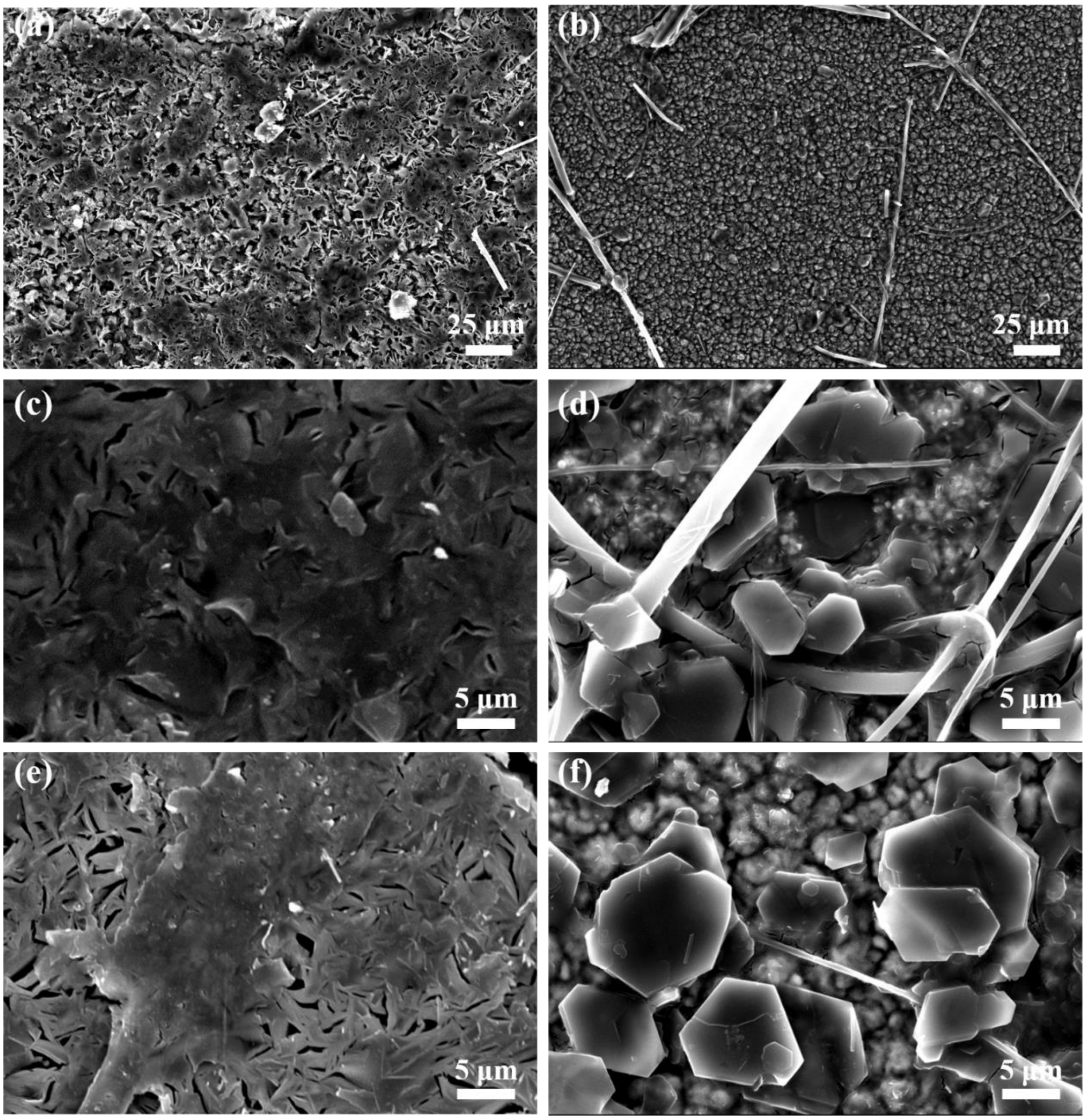

Figure S3. Scanning electron microscopy images of (a,c,e) CF and (b,d,f) Cu foil electrodes at the deposition current density of 5 mA cm^{-2} and deposition capacities of 2 mAh cm^{-2} with different cycles: (a) and (b) 20 cycles; (c) and (d) 50 cycles; (e) and (f) 80 cycles.

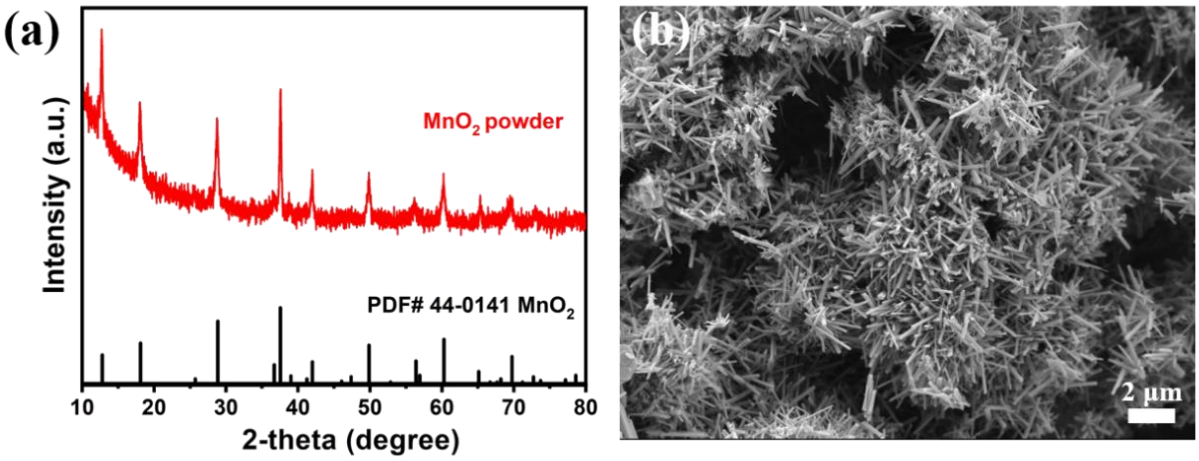

Figure S4. (a) XRD patterns and (b) SEM image of α-MnO_2.

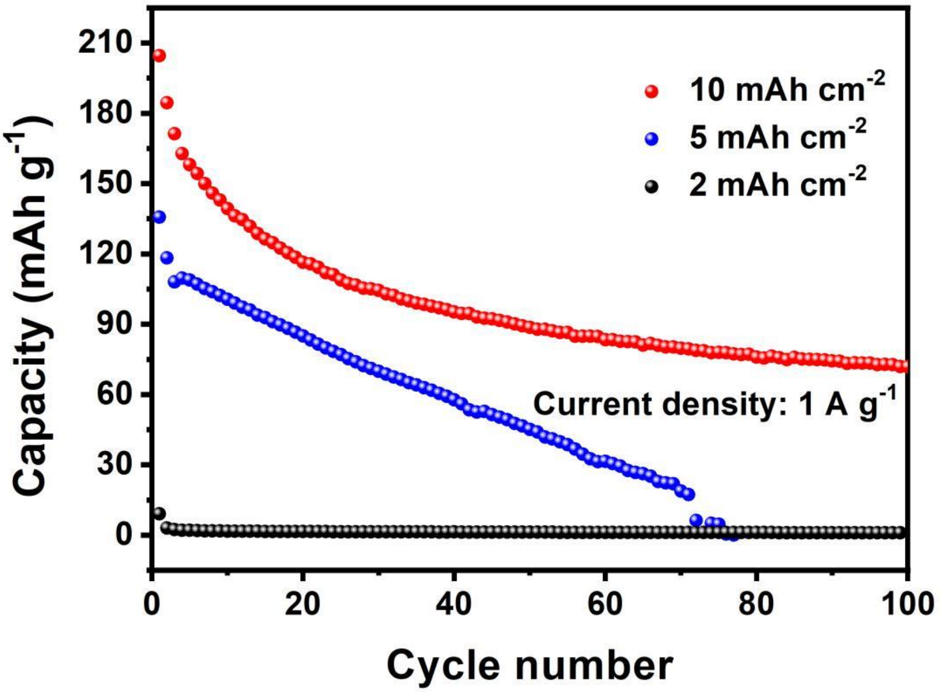

Figure S5. Cyclic performance of pre-deposited CF with different amounts of Zn (2 mAh cm^{-2}, 5 mAh cm^{-2} and 10 mAh cm^{-2}) in full cells.

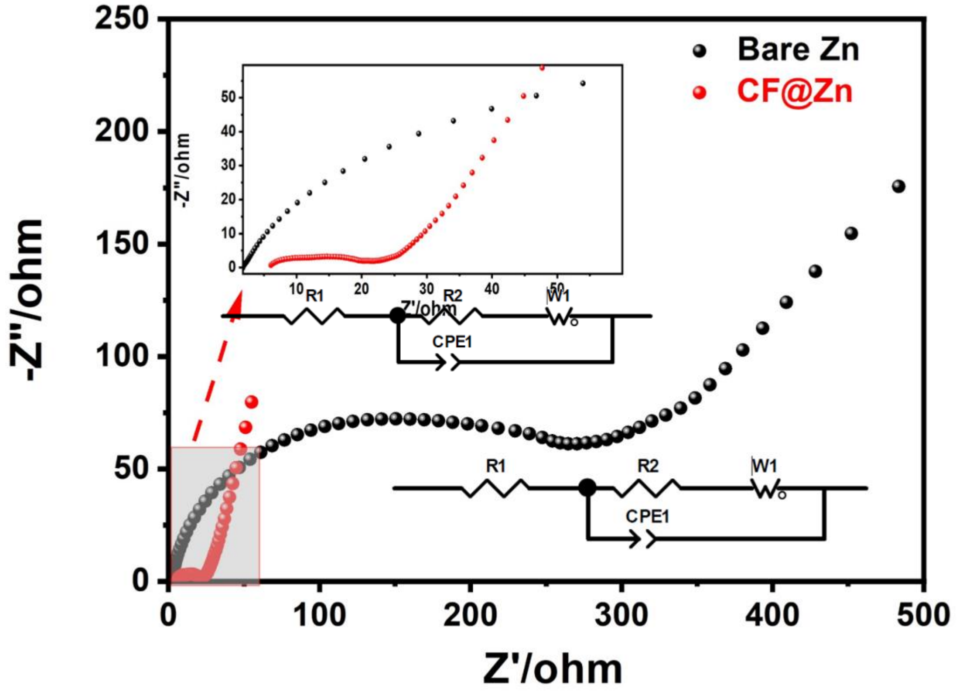

Figure S6. Nyquist plots of bare Zn and CF@Zn full cells at 1 A g^{-1} after 100 cycles (inset is the equivalent circuit).

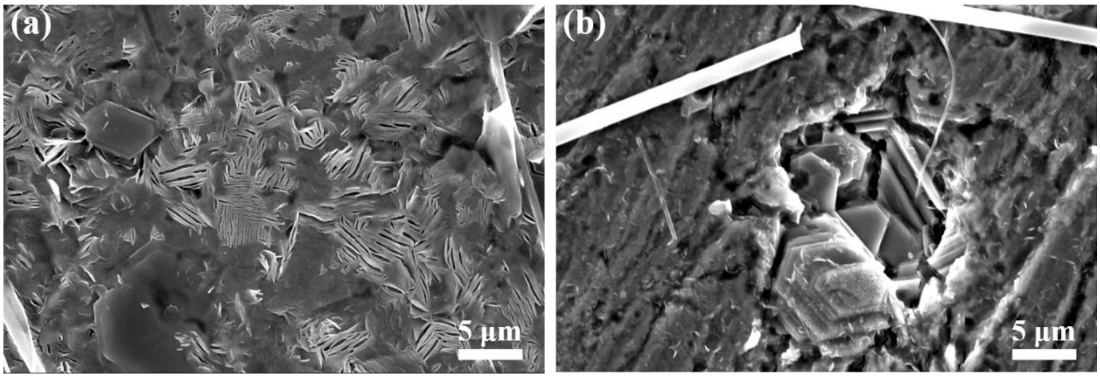

Figure S7. SEM images of a) CF@Zn and b) bare Zn anodes in full cells with α-MnO_2 cathode after 100 cycles at 1 A g^{-1}.

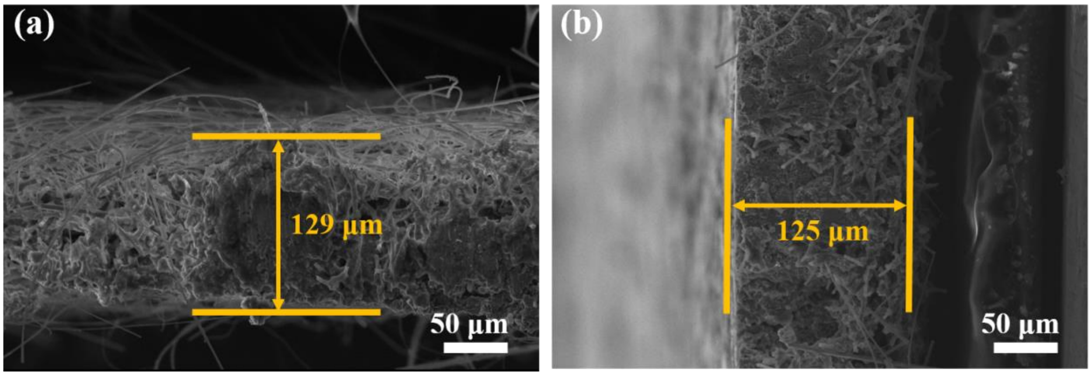

Figure S8. Cross-sectional view SEM images of a) CF@Zn and b) bare Zn anodes in full cells with α-MnO_2 cathode after 100 cycles at 1 A g^{-1}.

Table S1. A survey of zinc anodes with carbon-based hosts or modified by carbon-based materials and corresponding electrochemical properties.

**Zinc anodes**	**Current density (mA cm^{-2})**	**Areal Capacity (mAh cm^{-2})**	**Voltage hysteresis (V)**	**Worked time (h)**	**Reference**
rGO@Zn	2	2	0.1	200	[37]
graphite-coated Zn anode	0.1	0.1	0.028	200	[38]
Zn/C_3N_4	2	2	/	500	[39]
ZF@CB-NFC	0.5	0.5	0.160	400	[40]
CF@Zn	1	1	0.032	800	This work
	2	1	0.047	700	
	4	2	0.054	500	

Table S2. Different host materials for zinc metal anodes and the corresponding average CE value.

Skeletons	Current density (mA cm^{-2})	Areal Capacity (mAh cm^{-2})	Average Coulombic Efficiency	Reference
$Ti_3C_2T_x$ MXene	1	1	94.13%	[41]
Graphite felt	1	1	96.5%	[42]
Cu foil	5	2	83.1%	This work
CF	5	2	93.25%	This work

Table S3. The impedance parameters for full cells with α-MnO_2 cathode before cycling.

**Anode**	**R_s (Ω)**	**R_{ct} (Ω)**
CF@Zn	4.507	5.671
Zn	5.145	102

Table S4. The impedance parameters for full cells with α-MnO_2 cathode at 1 A g^{-1} after 100 cycles.

**Anode**	**R_s (Ω)**	**R_{ct} (Ω)**
CF@Zn	4.61	15.86
Zn	5.39	225.9

References

37. Shen, C.; Li, X.; Li, N.; Xie, K.; Wang, J.-g.; Liu, X.; Wei, B. Graphene-Boosted, High-Performance Aqueous Zn-Ion Battery. *Acs Appl. Mater. Inter.* **2018**, *10*, 25446-25453, doi:10.1021/acsami.8b07781.
38. Li, Z.; Wu, L.; Dong, S.; Xu, T.; Li, S.; An, Y.; Jiang, J.; Zhang, X. Pencil Drawing Stable Interface for Reversible and Durable Aqueous Zinc-Ion Batteries. *Adv. Funct. Mater.* **2021**, *31*, 2006495, doi:10.1002/adfm.202006495.
39. Liu, P.; Zhang, Z.; Hao, R.; Huang, Y.; Liu, W.; Tan, Y.; Li, P.; Yan, J.; Liu, K. Ultra-highly stable zinc metal anode via 3D-printed g-C3N4 modulating interface for long life energy storage systems. *Chem. Eng. J.* **2021**, *403*, 126425, doi:10.1016/j.cej.2020.126425.
40. Wang, A.; Zhou, W.; Huang, A.; Chen, M.; Chen, J.; Tian, Q.; Xu, J. Modifying the Zn anode with carbon black coating and nanofibrillated cellulose binder: A strategy to realize dendrite-free Zn-MnO2 batteries. *J. Colloid Interface Sci.* **2020**, *577*, 256-264, doi:10.1016/j.jcis.2020.05.102.
41. Tian, Y.; An, Y.; Wei, C.; Xi, B.; Xiong, S.; Feng, J.; Qian, Y. Flexible and Free-Standing Ti3C2Tx MXene@Zn Paper for Dendrite-Free Aqueous Zinc Metal Batteries and Nonaqueous Lithium Metal Batteries. *ACS Nano* **2019**, *13*, 11676-11685, doi:10.1021/acsnano.9b05599.
42. Wang, L.-P.; Li, N.-W.; Wang, T.-S.; Yin, Y.-X.; Guo, Y.-G.; Wang, C.-R. Conductive graphite fiber as a stable host for zinc metal anodes. *Electrochim. Acta* **2017**, *244*, 172-177, doi:10.1016/j.electacta.2017.05.072.
